# Daytime melatonin and light independently affect human alertness and body temperature

**DOI:** 10.1111/jpi.12583

**Published:** 2019-05-09

**Authors:** Renske Lok, Minke J. van Koningsveld, Marijke C. M. Gordijn, Domien G. M. Beersma, Roelof A. Hut

**Affiliations:** ^1^ Chronobiology Unit, Groningen Institute for Evolutionary Life Sciences University of Groningen Groningen The Netherlands; ^2^ Chrono@Work Groningen The Netherlands

**Keywords:** alertness, body temperature regulation, daytime, human, light, melatonin, placebo

## Abstract

Light significantly improves alertness during the night (Cajochen, *Sleep Med Rev*, 11, 2007 and 453; Ruger et al., *AJP Regul Integr Comp Physiol*, 290, 2005 and R1413), but results are less conclusive at daytime (Lok et al., *J Biol Rhythms*, 33, 2018 and 589). Melatonin and core body temperature levels at those times of day may contribute to differences in alerting effects of light. In this experiment, the combined effect of daytime exogenous melatonin administration and light intensity on alertness, body temperature, and skin temperature was studied. The goal was to assess whether (a) alerting effects of light are melatonin dependent, (b) soporific effects of melatonin are mediated via the thermoregulatory system, and (c) light can improve alertness after melatonin‐induced sleepiness during daytime. 10 subjects (5 females, 5 males) received melatonin (5 mg) in dim (10 lux) and, on a separate occasion, in bright polychromatic white light (2000 lux). In addition, they received placebo both under dim and bright light conditions. Subjects participated in all four conditions in a balanced order, yielding a balanced within‐subject design, lasting from noon to 04:00 pm. Alertness and performance were assessed half hourly, while body temperature and skin temperature were measured continuously. Saliva samples to detect melatonin concentrations were collected half hourly. Melatonin administration increased melatonin concentrations in all subjects. Subjective sleepiness and distal skin temperature increased after melatonin ingestion. Bright light exposure after melatonin administration did not change subjective alertness scores, but body temperature and proximal skin temperature increased, while distal skin temperature decreased. Light exposure did not significantly affect these parameters in the placebo condition. These results indicate that (a) exogenous melatonin administration during daytime increases subjective sleepiness, confirming a role for melatonin in sleepiness regulation, (b) bright light exposure after melatonin ingestion significantly affected thermoregulatory parameters without altering subjective sleepiness, therefore temperature changes seem nonessential for melatonin‐induced sleepiness, (c) subjective sleepiness was increased by melatonin ingestion, but bright light administration was not able to improve melatonin‐induced sleepiness feelings nor performance. Other (physiological) factors may therefore contribute to differences in alerting effects of light during daytime and nighttime.

## 1 INTRODUCTION

The suprachiasmatic nucleus (SCN) is the pacemaker of the mammalian circadian timing system. Many physiological rhythms are regulated by the SCN, like melatonin secretion and core body temperature.^1^, ^2^ Plasma melatonin rises during the evening and peaks at night, while levels are virtually zero during daytime in most people.^3^ Core body temperature (CBT) peaks in the evening and has its nadir in the early morning.^4^ Correlations between patterns of melatonin and CBT have been shown.^5^, ^6^ Alertness, which is associated with high levels of environmental awareness, also fluctuates in a circadian manner,^7^ with lowest levels in the early morning, and high levels during daytime. Mutual regulatory relationships have been suggested between melatonin, CBT, and alertness.^6^ Light‐induced melatonin suppression is associated with decreased sleepiness,^8^, ^9^ although probably not at intermediate indoor light levels.^10^, ^11^ Melatonin ingestion increases subjective sleepiness and lowers CBT,^12^ but CBT manipulations affect subjective sleepiness at times of day when melatonin is virtually absent, suggesting a relationship between CBT and sleepiness independent of melatonin.^13^

Alertness is known to affect many functions, such as performance, psychological and physiological well‐being, caloric intake, and pain sensitivity.^14^, ^15^, ^16^, ^17^ Displaying optimal alertness is thus beneficial, and many studies have attempted to improve alertness using monochromatic or polychromatic light (for review see,^18^, ^19^). Exposure to light improves alertness during nighttime, when melatonin concentrations are usually high and CBT decreases.^9^, ^10^, ^11^, ^16^, ^20^, ^21^, ^22^, ^23^, ^24^, ^25^ However, several studies report absence of light‐induced alertness during daytime, when alertness and CBT levels are high and melatonin is absent,^18^, ^20^, ^26^, ^27^, ^28^, ^29^, ^30^, ^31^, ^32^, ^33^, ^34^, ^35^, ^36^, ^37^, ^38^, ^39^, ^40^, although other studies report contradictory data.^10^, ^41^, ^42^ These paradoxical findings can be reconciled when an alertness ceiling level during daytime is considered, which prevents light to further increase alertness. Differences in melatonin and CBT levels might contribute to these dissimilarities in light‐induced alertness.

In the Netherlands, melatonin is sold over the counter up until 5 mg units for self‐treatment of sleep and jet‐lag problems.^43^, ^44^ and to induce circadian phase shifts.^45^, ^46^ Administration of varying concentrations of exogenous melatonin has resulted in increased levels of subjective sleepiness, coinciding with decreased CBT, which is possibly posture‐dependent.^12^, ^47^ Sleepiness inducing effects of melatonin have been attributed to its ability to pass the blood‐brain barrier and affect hypothalamic CBT regulation.^48^

Exogenous melatonin decreases alertness, but its concentration is unaffected by light. By studying the combined effects of melatonin and light on alertness, CBT, and skin temperature regulation, it can be assessed whether (a) alerting effects of light are melatonin suppression dependent, (b) soporific effects of melatonin are mediated via the thermoregulatory system, and (c) light‐induced alertness depends on melatonin‐induced sleepiness levels during daytime. The goal of this experiment was therefore to investigate these underlying relationships using exogenous melatonin and bright light exposure in combination.

## 2 MATERIALS & METHODS

Subjects (5 female, 5 male) were recruited via posters and flyers distributed within the University of Groningen and mouth‐to‐mouth advertisement. Participants aged 20‐30 years gave written informed consent and received financial compensation for participation. The protocol, questionnaires, and consent forms were approved by the medical ethics committee of the University Medical Center Groningen (NL61863.042) and were in agreement with the Declaration of Helsinki (2013). All participants were students, reported no health problems (assessed via an in‐house developed general health questionnaire), were intermediate chronotypes (Munich Chronotype Questionnaire,^49^ mid‐sleep on free days corrected for sleep debt on work days (MSF_sc_) between 3.88 and 6.17), and did not report sleep problems (Pittsburgh Sleep Quality Index < 6.^50^). Exclusion criteria were as follows: (a) chronic medical conditions or the need for (sleep) medication use, including melatonin, (b) shift work 3 months before participation, (c) having travelled over multiple time‐zones within 2 months before participation, (d) smoking, (e) excessive use of alcohol (>3 consumptions per day), (f) use of (recreational) drugs in the last year, (g) a body mass index outside the range of 18‐27, and (h) inability to complete Ishihara color blindness test^51^ without errors. Participant characteristics are described in Table S1.

**Figure 1:**
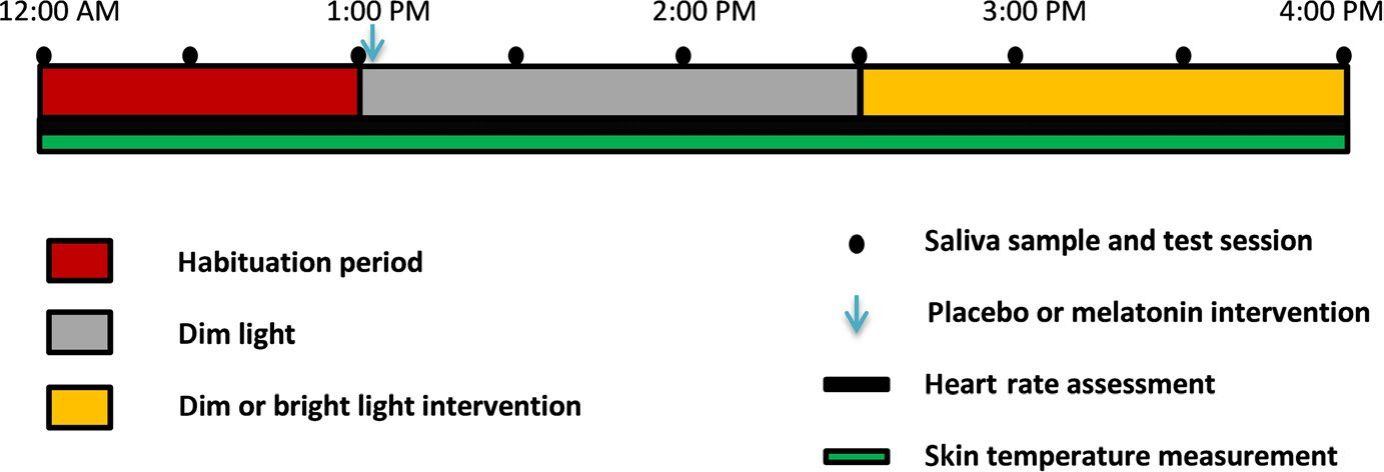
Schematic representation of the experimental design. The protocol lasted from 12:00 am to 04:00 pm and comprised of an hour of habituation, followed by the placebo or melatonin intervention and subsequent dim or bright light exposure

The experiment was conducted in April and May 2018, with local time expressed as GMT + 2. Subjects arrived at the human isolation facility of the University of Groningen at 11:30 am, where they stayed in individual rooms in dim light (DL, Figure 1). Participants were equipped with DS1922L Ibuttons (Thermochron) for measuring skin temperature on the forehead (*T*_forehead_), navel (*T*_navel_), right and left subclavicular regions (*T*_subclavicular_), hand palms (*T*_hands_), underneath the feet (*T*_feet_), and in the pulp of the first toe (*T*_toes_). An Actiheart monitor (CamNTech) was attached to the left chest to measure heart rate. Participants were seated at a desk in a semi‐recumbent position. The protocol started with 1 hour of habituation. At 01:00 pm, participants were given either a placebo (empty gelatin capsule) or melatonin pill (identical gelatin capsule filled with 5 mg time release melatonin, Melatomatine; Vemedia). 90 minutes after placebo or melatonin intervention, participants either completed the experiment in DL or were exposed to bright light (BL) for 90 minutes. Test sessions to assess alertness were completed half hourly from 12:00 pm onwards. Each participant received the placebo and melatonin intervention in DL and BL and therefore participated four times. Conditions were imposed in balanced order and participation was separated by at least 1 week. Subjects were instructed to maintain a stable sleep‐wake rhythm throughout the experiment and were reminded of this 2 days before participation. Subjects participated on the same day of the week and were blinded to the melatonin or placebo intervention, rendering a balanced, single‐blind within‐subject design where subjects were their own control.

At the start of a test session, saliva was collected using Sarstedt Salivettes with a cotton swab (Sarstedt BV). Subsequently, participants placed a CBT pill (e‐Celsius Performance, BodyCap, 60‐second sampling interval with ±0.2°C accuracy and 0.1°C variability level) under the tongue to measure temperature underneath the tongue (*T*_tongue_). Participants completed the Karolinska Sleepiness Scale (KSS),^52^ followed by a 5‐minute auditory psychomotor vigilance task (PVT,^53^ E‐prime version EP2Pro2.0.10.242). After completion, participants removed the CBT pill. Skin temperature (60 seconds sampling interval, 0.0625°C resolution, and 0.5°C accuracy) and heart rate (15 seconds sampling interval) were measured continuously.

Dim and bright polychromatic white light was delivered by a modified Philips Energy Up light (HF3419/02; Philips, Figure S1 and Table S2). The light was placed at a distance of 20 cm, generating 2000 lux (BL) or 10 lux (DL, by covering the lamp with neutral density filters) measured at eye level.

For every individual, PVT anticipation errors occurred when a reaction time was shorter than the average of all test sessions ‐ two standard deviations, and omission errors occurred when a reaction time exceeded the average reaction time of all test sessions + two standard deviations. Skin temperature outliers, defined as values where absolute consecutive temperature change exceeding 2°C, were omitted. Distal (*T*_distal_) skin temperature was calculated as the average temperature of *T*_hands_ and *T*_feet_. Proximal skin temperature (*T*_proximal_) was defined as *T*_subclacivular_, and the distal‐proximal gradient (*T*_DPG_) was calculated as *T*_distal_ minus *T*_proximal_.^54^ Data of heart rate measurement was analyzed using Actiheart software (version 4.0.116) and can be found in Figure S2. Results of *T*_tongue_ measurements were averaged per test session when temperatures reached an asymptote.

All data were z‐transformed per individual. To assess effects of melatonin administration on alertness, data after melatonin or placebo administration were expressed relative to measurements at 01:00 pm. To determine effects of the light intervention, data collected during the last three test sessions were expressed relative to data collected at 2:30 pm. Skin temperature data were averaged over 5 minutes prior to both interventions. A 5‐minute running average was calculated to smooth short‐term fluctuations. Saliva samples were stored at −20°C until analysis, when samples were defrosted and diluted, ranging from 0 to 500 times, resulting in values within the range of the standard assay curve. Dilution curves were tested for linearity. Radioimmunoassay (RIA) analysis (RK‐DSM2; Bühlmann Laboratories AG) was used to determine melatonin concentrations (0.5 pg/mL detection limit, 13.1% intra‐assay variation).

Linear models were constructed in RStudio (version 1.0.136) with subjective sleepiness, PVT performance, *T*_tongue_, skin temperature, and heart rate measurements as dependent variables. Continuous data were grouped in half‐hourly bins. Independent variables were time of day and intervention. Fixed effects consisted of time of day (as categorical variable), intervention, and the interaction term. Critical two‐sided significance level alpha was 0.05 for all statistical tests.

## 3 RESULTS

**Figure 2:**
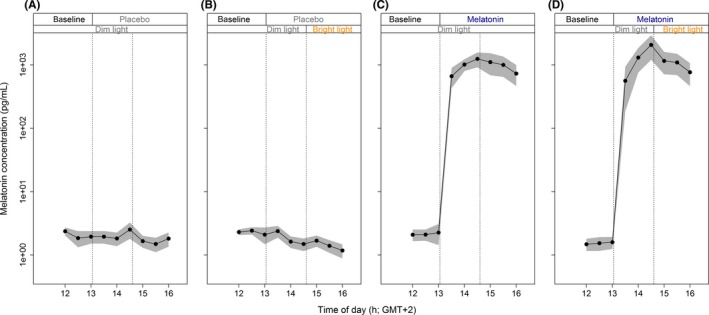
Time course of melatonin concentrations. Concentrations were determined by half‐hourly saliva samples, before and after placebo/melatonin administration. All data represent mean (black dots) ± standard error of the mean (gray), N = 10 per group

Administration of exogenous melatonin resulted in increased salivary melatonin concentrations (*F*_1,114_ = 11.55, *P* = 1.26e‐6), which peaked approximately 1.5 hours after administration (Figure 2C and 2D, see Figure S3 for individual data). Bright light exposure did not alter melatonin concentrations, neither after placebo (*F*_1,49_ = 0.63, *P* = 0.43) nor melatonin (*F*_1,49_ = 0.042, *P* = 0.84) ingestion. Melatonin levels decreased to preadministration levels before the start of the light intervention in one subject, this subject was therefore excluded from determining light intervention effects.

**Figure 3:**
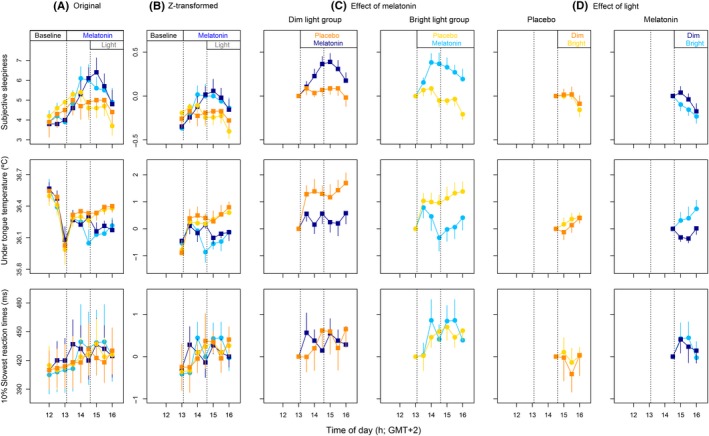
Effects of melatonin and light on sleepiness, *T*_tongue,_ and slowest reaction times. (A) Original data. (B) Z‐transformed data. (C) Effects of melatonin in the dim and bright light group. (D) Effects of light during the interval after placebo or melatonin administration. Data in C and D data are expressed relative to values at 13:00. E and F data are expressed relative to values at 14:30. DL data are depicted in orange (placebo) and dark blue (melatonin). BL data are yellow (placebo) and light blue (melatonin). All data represent mean ± standard error of the mean, N = 10 per group, except for panel D, in which N = 9 per group

In the first 90 minutes after melatonin administration, there was a significant increase in subjective sleepiness, without a significant change in *T*_tongue_ and performance (Figure 3C and Table 1). Bright light exposure did not affect performance and subjective sleepiness after placebo or melatonin ingestion, nor did it affect *T*_tongue _after placebo ingestion. After melatonin administration however, BL exposure significantly increased *T*_tongue_ (Figure 3D and Table 1). There were no significant effects of melatonin or light administration on other output measures of performance, such as anticipation‐ or omission errors (*P* > 0.05, data not shown).

**Table 1:** Statics of melatonin and light effects on sleepiness, *T*_tongue,_ and performance. Values from linear mixed models on z‐transformed data

	*Df*	Effect of melatonin		*Df*	Effect of light after placebo ingestion		Effect of light after melatonin ingestion	
		*F*	*P*		*F*	*P*	*F*	*P*
Subjective sleepiness	1,114	114	**<0.001**	1,49	0.94	0.33	0.10	0.75
*T*_tongue_	1,114	1.27	0.29	1,49	0.22	0.64	7.77	**0.008**
Performance	1,114	0.42	0.73	1,49	1.99	0.30	1.10	0.30

*P*‐values that reached significant are indicated in bold for optimal clarity

**Figure 4:**
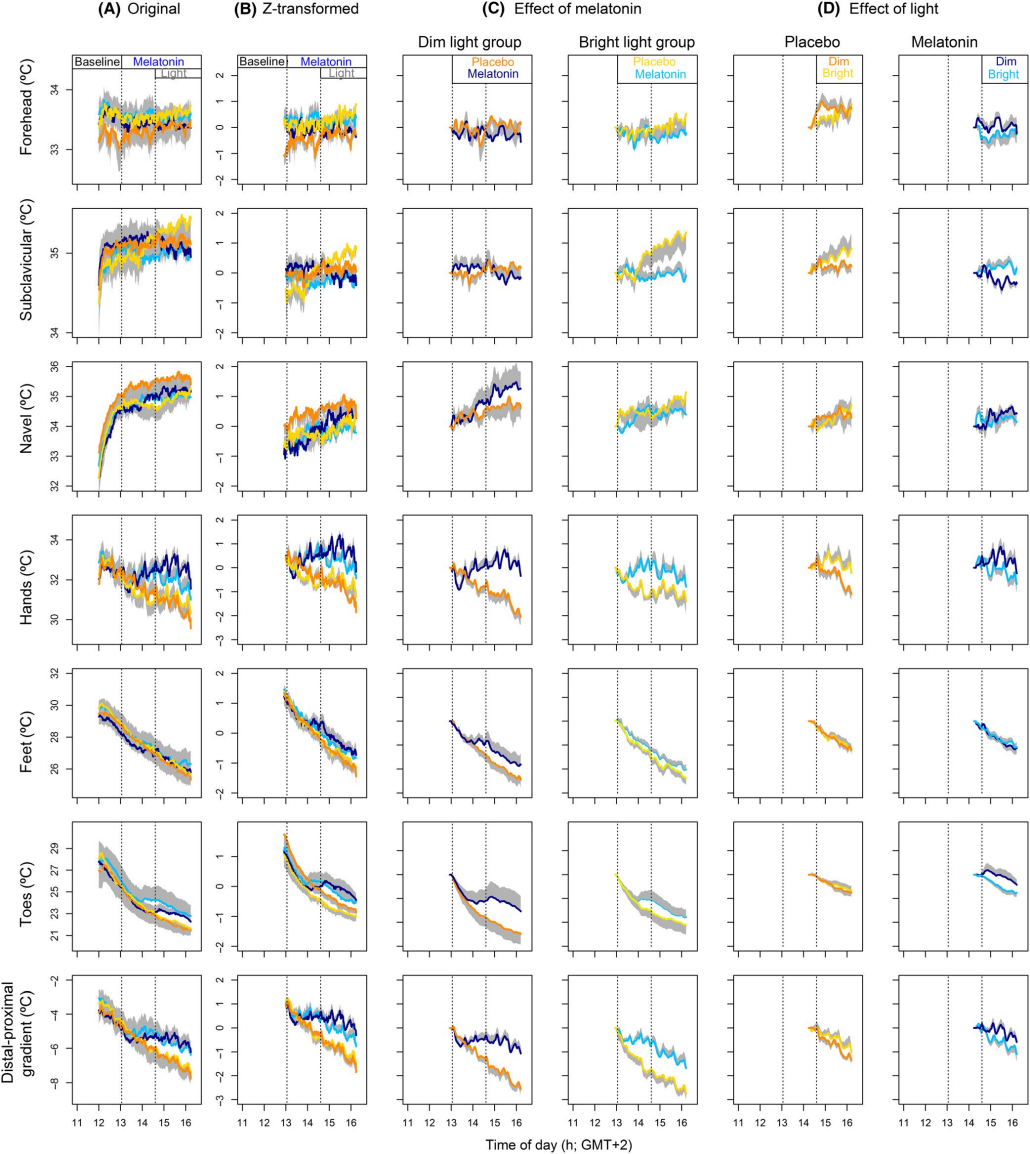
Effects of melatonin and light on skin temperatures. (A) Original data. (B) Z‐transformed data. (C) Effects of melatonin in the dim and bright light group. (D) Effects of light during the interval after placebo or melatonin administration. Data in C and D data are expressed relative to values at 13:00. E and F data are expressed relative to values at 14:30. DL data are depicted in orange (placebo) and dark blue (melatonin). BL data are in yellow (placebo) and light blue (melatonin). All data represent mean ± standard error of the mean, N = 10 per group, except for panel D, which includes N = 9 subjects

Melatonin administration increased temperature measured in *T*_hands_ and *T*_DPG_ (Figure 4C and Table 2). Other skin temperature parameters, such as *T*_toes_, *T*_feet_, *T*_subclavicular_, *T*_navel_, and *T*_forehead_ were unaffected by melatonin administration. BL exposure after placebo ingestion significantly increased T_hands_ and T_DPG_, while T_distal_ and T_DPG_ were significantly decreased after melatonin administration, with an increase in T_proximal_.

**Table 2:** Statics of melatonin and light effects on skin temperatures. Values from linear mixed models on z‐transformed data

	*Df*	Effect of melatonin		*Df*	Effect of light after placebo ingestion		Effect of light after melatonin ingestion	
		*F*	*P*		*F*	*P*	*F*	*P*
*T*_forehead_	1,114	0.00	0.99	1,49	2.25	0.14	1.52	0.22
*T*_subclavicular_	1,114	1.99	0.12	1,49	2.21	0.14	9.93	**0.002**
*T*_navel_	1,114	0.14	0.94	1,49	0.00	0.95	0.02	0.88
*T*_hands_	1,114	4.83	**0.004**	1,49	10.22	**0.002**	3.94	**0.04**
*T*_feet_	1,114	0.64	0.56	1,49	0.17	0.68	0.00	0.98
*T*_toes_	1,114	0.51	0.67	1,49	0.55	0.46	8.30	**0.005**
*T*_DPG_	1,114	7.00	**<0.001**	1,49	6.30	**0.01**	6.75	**0.01**

*P*‐values that reached significant are indicated in bold for optimal clarity

## 4 DISCUSSION

Light induces significant improvements in alertness during nighttime, but similar effects are difficult to demonstrate during the day. Differences in melatonin and core body temperature levels at those times of day might contribute to different responses in alertness. The goal of this experiment was to assess whether (a) alerting effects of light are melatonin suppression dependent, (b) these effects were mediated via the thermoregulatory system, and (c) light‐induced alertness depended on (melatonin‐induced) sleepiness levels during daytime. Melatonin administration during daytime increased salivary melatonin concentrations to supra‐pharmacological levels, which coincided with decreased subjective alertness, implicating a role for melatonin in alertness regulation. Decreased alertness was paralleled by increased *T*_distal_ and *T*_DPG_, indicating a role for thermoregulation in sleepiness inducing effects of melatonin. However, bright light exposure after melatonin ingestion increased *T*_proximal_ and *T*_tongue_ and decreased *T*_distal_, suggesting that light is able to counteract thermoregulatory effects of exogenous melatonin. Importantly, subjective sleepiness was not significantly altered by bright light exposure. Thermoregulatory processes therefore seem not directly related to alerting effects of light. Subjective sleepiness was increased by melatonin ingestion, but bright light administration did not affect subjective sleepiness nor performance; therefore, light cannot restore melatonin‐induced sleepiness.

In our subjects, 5 mg melatonin administration resulted in peak levels between 600 and 8000 pg/mL, leading to levels 30‐400 times higher than maximum endogenous concentrations.^55^ Underlying relationships between melatonin, alertness, and temperature suggested here may therefore not reflect regulatory relationships under natural circumstances. However, exogenous melatonin was necessary to investigate underlying relationships. Relatively high concentrations were applied to maximize chances of significant results. Although salivary melatonin levels were high throughout the experiment, we cannot exclude the possibility that some of the effects reported here were also mediated through metabolites of melatonin, which include *N*(1)‐acetyl‐*N*(2)‐formyl‐5‐methoxykynuramine (AFMK), *N*(1)‐acetyl‐5‐methoxy‐kynuramine (AMK), 6‐hydroxymelatonin, and 6‐sulfatoxy melatonin.^56^ The current experiment reveals acute effects of melatonin and light on alertness independent of clock manipulations, since both melatonin and light were administrated during relatively unresponsive parts of the respective human phase response curves.^57^, ^58^

Regulatory relationships between melatonin and alertness have been suggested.^8^, ^47^, ^59^, ^60^, ^61^  The current study shows that exogenous melatonin during daytime leads to a significant increase in subjective sleepiness, with peaks coinciding with the peak in salivary melatonin concentration. Melatonin receptors have been found in the cortex and thalamus, which are areas that have been associated with hypnotic effects of melatonin.^62^, ^63^, ^64^ Endogenous melatonin production only occurs during darkness, indicating that in diurnal animals, melatonin may signal the optimal time for sleep^65^ and therefore induces sleepiness.^66^, ^67^ One of the distinct functions of melatonin in humans is priming of sleep‐associated brain activation patterns,^67^ as it provides information about environmental light conditions and time of day.^66^ The relatively fast sleepiness inducing effects of melatonin suggest that this might be a direct physiological consequence*,* 68 independent of melatonin's action as zeitgeber signal***.*** 69, 70

There are multiple mechanisms by which exogenous melatonin can influence sleepiness. Melatonin is highly lipophilic, and it easily passes the blood‐brain barrier.^48^ Binding sites for melatonin have been found in hypothalamic areas associated with thermoregulatory processes.^64^, ^71^, ^72^ Secretion of melatonin, modulated by the SCN, influences CBT levels.^73^, ^74^ Heat (re)distribution from the core to extremities occurs by blood transport through proximal and distal skin regions. MT‐1 melatonin receptors, with vasoconstrictive properties, are located in precapillary smooth muscles in proximal and distal skin regions while MT2‐receptors, which cause vasodilation, are located in arteriovenous anastomoses (AVAs) in distal skin regions.^75^, ^76^, ^77^ Melatonin might therefore also affect skin temperature locally. Since melatonin affects temperature regulation on multiple levels and changes in body temperature correlate to altered alertness, it has been suggested that melatonin induces sleepiness via influencing body temperature.^78^ In our experiment, melatonin administration did not result in a significant decrease in *T*_tongue_, although subjective sleepiness increased. However, it has been claimed that subjective sleepiness is mainly influenced by changes in *T*_distal_.^76^ In our study, *T*_distal_ increase was paralleled by increased subjective sleepiness, indicating that melatonin may indeed induce sleepiness through thermoregulatory effects. Temperature effects after BL exposure occurred without significantly altering subjective sleepiness scores, indicating that soporific effects of melatonin may not depend on thermoregulation after all, as has been indicated by others.^79^ A decrease of 1°C in *T*_distal _has been reported to significantly decrease subjective sleepiness,^76^ while light decreased *T*_distal_ with approximately 0.5°C in our study. It is therefore possible that temperature changes due to light exposure were not extensive enough to change sleepiness.

Our study did not demonstrate alerting effects of light after placebo ingestion, indicating that light under these circumstance does not induce alertness during daytime, confirming previous observations.^33^ This emphasizes differences between night and day, since alerting effects of light during the night have been reported (reviews in Ref.^18^, ^19^). A possible explanation is based on high levels of daytime alertness, causing a possible ceiling.^80^ Exogenous melatonin ingestion was able to increase subjective sleepiness, removing a potential ceiling of maximal alertness during daytime and allowing for alertness improvement by light. Since there were no significant effects of light on feelings of alertness, even after inducing additional sleepiness by exogenous melatonin, it is possible that (a) melatonin might not have reduced alertness sufficiently, therefore the ceiling of alertness might not have decreased sufficiently for alerting effects of light to occur, (b) a ceiling effect does not explain the lack of alerting effects of light during daytime, or (c) light cannot induce alertness when melatonin concentrations are high. Since subjective sleepiness scores after melatonin administration are similar to those reported during the late evening, when alerting effects of light have been found,^21^ it is unlikely that ceiling effects explain discrepancies between night and day. Imaging studies have shown clear differences in brain activity patterns between melatonin‐ and sleep deprivation‐induced sleepiness.^67^ Melatonin administration induces changes in brain activity patterns resembling patterns seen during sleep, while sleep deprivation does not. Underlying neuronal mechanisms therefore differ. Studies investigating alerting effects of light during daytime after sleep deprivation found significant effects^41^ while several studies in rested individuals did not for a review see.^18^, ^19^ Sleep deprivation alters sleep pressure, suggesting that these levels are important for alerting effects of light.^67^ Alerting effects of light have been found after exogenous melatonin administration in the evening, indicating that light can induce alertness while melatonin levels are high, supporting the notion that other (physiological) factors, such as sleep pressure, might be of importance for alerting effects of light.^81^ Although subjective measures and most physiological measures reflect a significant increase in sleepiness, performance remained unaffected by both melatonin and light intervention. This is in contrast with other studies, which report a decreased performance after oral melatonin administration.^59^, ^68^, ^82^, ^83^ Cognitive performance tasks might not always accurately reflect alertness^19^, ^84^ since performance does not correlate with validated subjective measures of alertness or temperature parameters that are associated with sleepiness. Correlations between subjective alertness and physiological measures of alertness might even be stronger compared to the relationship with performance tasks.^85^

In conclusion, 5 mg oral melatonin ingestion during daytime causes a significant increase in distal skin temperature and subjective sleepiness, without affecting performance. Bright light exposure after melatonin ingestion does not reduce subjective sleepiness, but does increase body temperature and proximal skin temperature, while decreasing distal skin temperature. Melatonin is unlikely to induce sleepiness via the thermoregulatory system, since temperature levels can be restored without affecting alertness. Through which mechanisms melatonin does induce sleepiness, remains unknown. As alerting effects of light after exogenous melatonin administration have been determined in the evening, dissimilarities in alerting effects of light between night and day might be caused by different mechanisms underlying feelings of sleepiness, possibly due to differences in (physiological) parameters, such as sleep pressure levels.

## CONFLICT OF INTEREST

The authors have declared the following potential conflict of interest with respect to the research, authorship, and/or publication of this article: Philips Drachten has made an in‐kind contribution to the experiment. Dr Gordijn reports receiving consultancy fees from Philips Consumer Lifestyle, not related to the submitted work.

## AUTHOR CONTRIBUTION

Author Lok has written the manuscript, while both Lok and van Koningsveld have been involved in data acquisition and analysis. Authors Gordijn, Beersma, and Hut contributed to the concept design, interpretation of data, and drafting of the manuscript.
