# Supplementary material for: Daytime melatonin and light independently affect human alertness and body temperature
Source: J Pineal Res. 2019 May 9;67(1):e12583. doi: 10.1111/jpi.12583 (PMC6767594; doi:10.1111/jpi.12583)
Supplement: Supplementary file 1 [file JPI-67-na-s001.docx]

**Title:** Daytime melatonin and light independently affect human alertness and body temperature.

**Running title:** Melatonin and light effects on temperature.

**Authors:** Renske Lok^1,3^, Minke J. van Koningsveld^1^, Marijke C.M. Gordijn^1,2^, Domien G.M. Beersma^1^, Roelof A. Hut^1^

**Contact Information: ^1^**Chronobiology unit, Groningen Institute for Evolutionary Life Sciences, University of Groningen, PO box 11103, 9700CC, Groningen, the Netherlands.

^2^Chrono@Work B.V. Friesestraatweg 213, 9743 AD Groningen, The Netherlands.

^3^To whom all correspondence should be addressed: Renske Lok, Chronobiology Unit, Groningen Institute for Evolutionary Life Sciences, University of Groningen, PO Box 11103, 9700CC, Groningen, The Netherlands; e-mail: renske.lok@rug.nl.

**Supplementary information**

**Table S1:** Participant characteristics

| **Characteristic** | **Mean ± SEM** |
| --- | --- |
| Age (y) | 23.20 ± 1.08 |
| MSF_sc_ | 4.77 ± 0.27 |
| PSQI | 3.00 ± 0.42 |
| Caffeine (cups) | 1.11 ± 0.35 |
| BMI (kg/m2) | 21.47 ± 0.37 |

**Table S2:** Photometric properties of background- and experimental light according to the Lucas file(Lucas et al., 2014)

| Type | Color temperature (K) | Illuminance (lux) | Cyanopic (lux) | Melanopic (lux) | Rhodopic  (lux) | Chloropic (lux) | Eyrthropic (lux) |
| --- | --- | --- | --- | --- | --- | --- | --- |
| Dim light | 5800 | 10 | 10.1 | 9.71 | 9.78 | 10.21 | 9.89 |
| Bright light | 5800 | 2000 | 1744 | 1933 | 1934 | 1975 | 1925 |

**
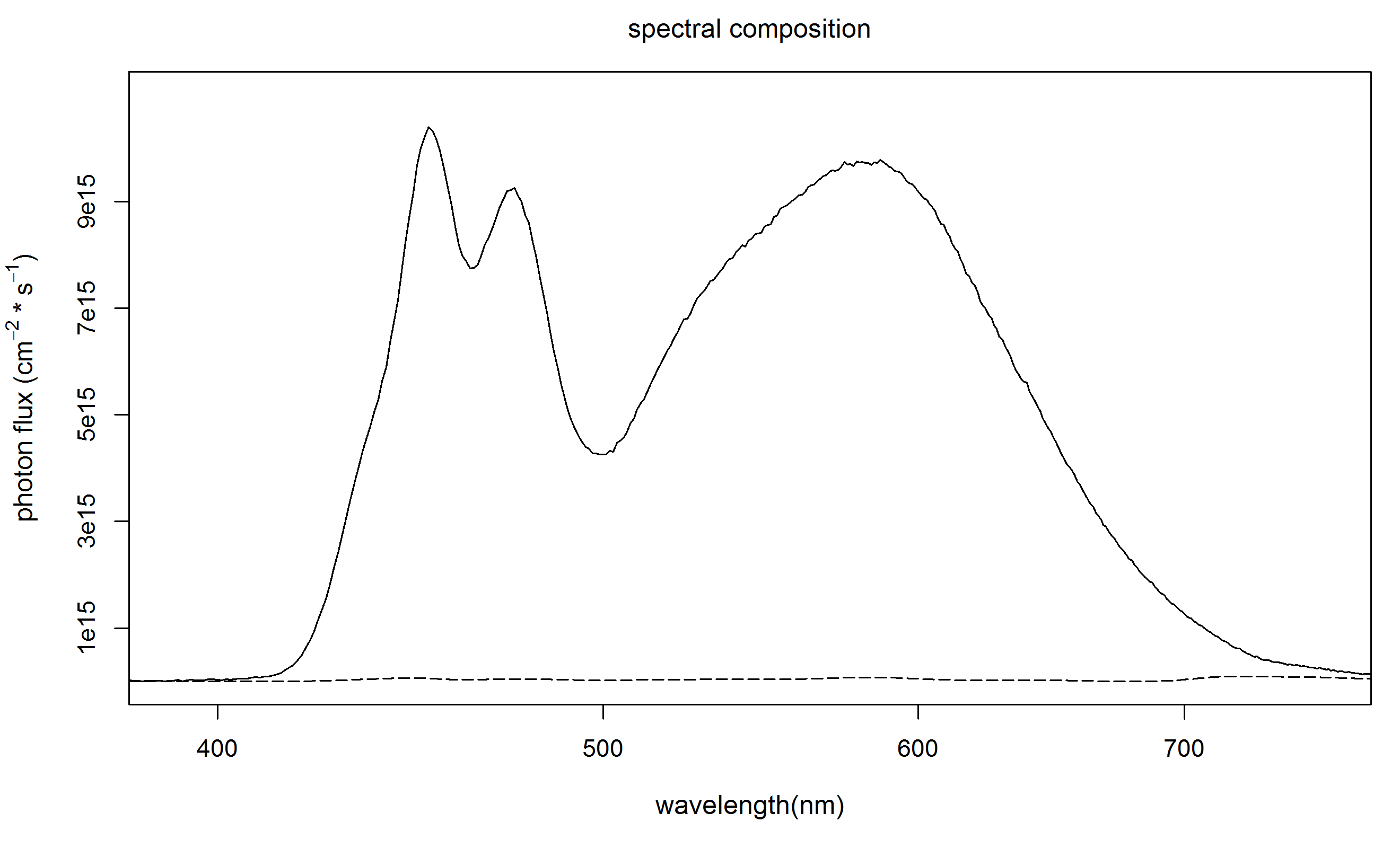
**

**Figure S1:** Spectral composition of dim- (dashed line) and bright light (solid line). Illuminance was measured on the vertical plane at the level of the eye. The light was generated with a modified Philips Energy Up light, in which two white LEDs had been substituted by two blue LEDs.

**
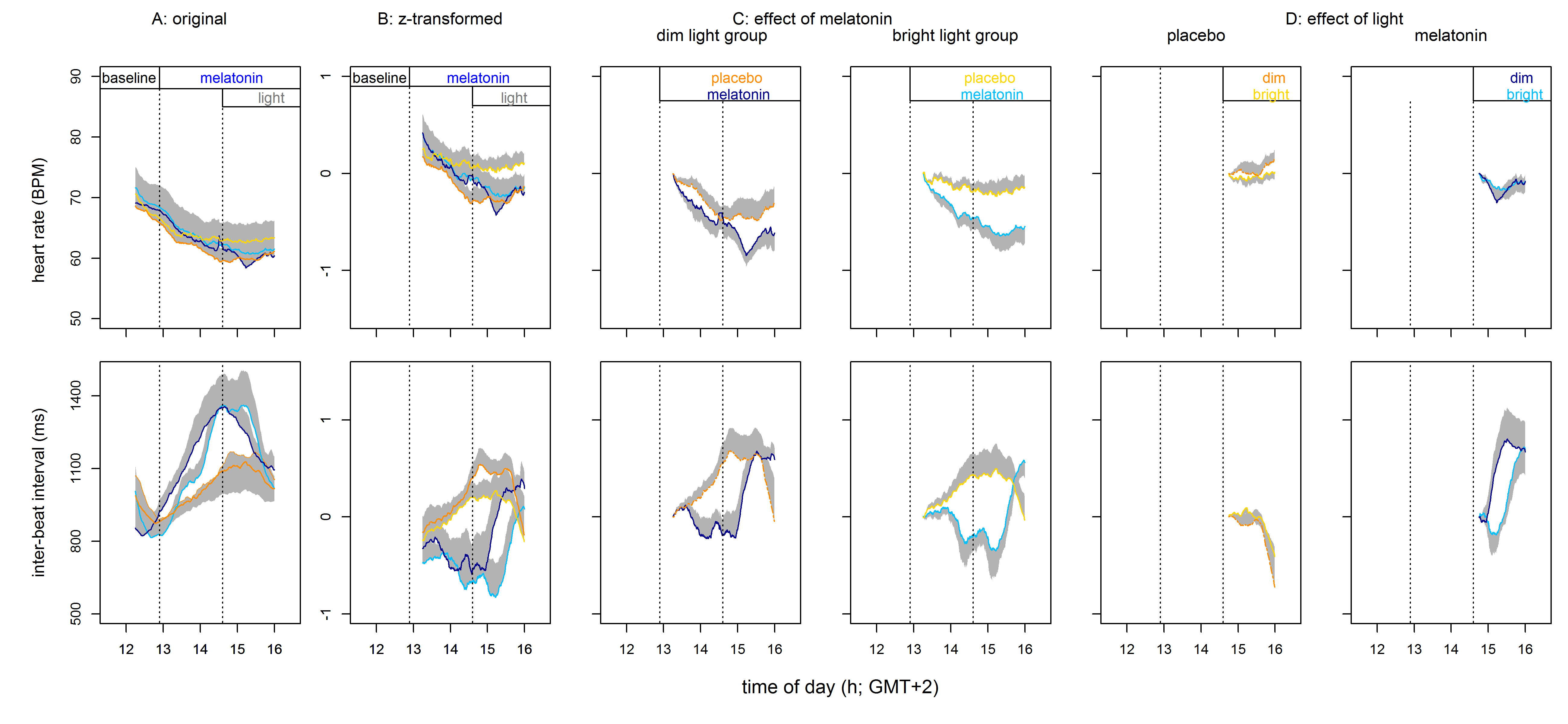
**

**Figure S2:** **Effects of melatonin and light on heartrate and inter-beat interval.** A) Original data. B) Z-transformed data. C) Effects of melatonin in the dim and bright light group. D) Effects of light during the interval after placebo or melatonin administration. Data in C and D data are expressed relative to values at 13:00. E and F data are expressed relative to values at 14:30. DL data are depicted in orange (placebo) and dark blue (melatonin), BL data are yellow (placebo) and light blue (melatonin). All data represent mean ± standard error of the mean, N=10 per group, except for panel D, in which N=9 per group.

**Table S3: Statics of melatonin and light effects on heartrate and inter-beat interval.** Values from linear mixed models on z-transformed

data.

|  |  | **Effect of melatonin under dim light** | | **Effect of melatonin**  **under bright light** | |  | **Effect of light after placebo ingestion** | | **Effect of light after melatonin ingestion** | |
| --- | --- | --- | --- | --- | --- | --- | --- | --- | --- | --- |
|  | *Df* | *F* | *p* | *F* | *p* | *Df* | *F* | *p* | *F* | *p* |
| Heartrate | 1,55 | 0.15 | 0.70 | 5.49 | **0.02*** | 1,49 | 0.08 | 0.77 | 0.81 | 0.37 |
| Inter-Beat Interval | 1,55 | 2.13 | 0.15 | 0.07 | 0.79 | 1,49 | 3.23 | 0.08 | 0.41 | 0.53 |


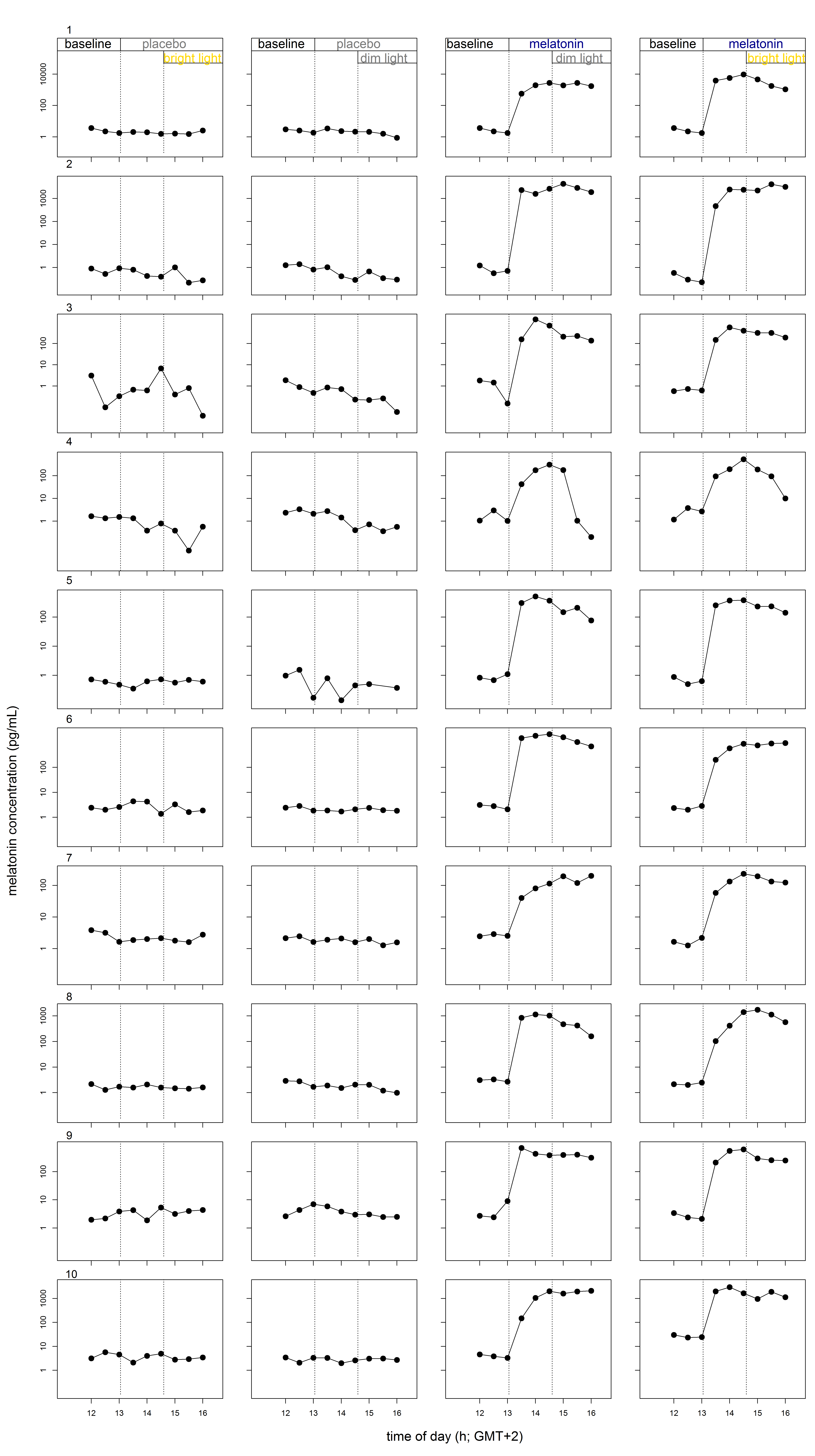


**Figure S3:** Individual data of melatonin concentrations of placebo/melatonin interventions plotted against time of day. Placebo/melatonin and dim/bright light administration is indicated by top panels. Individuals are numbered 1 through 10.

**
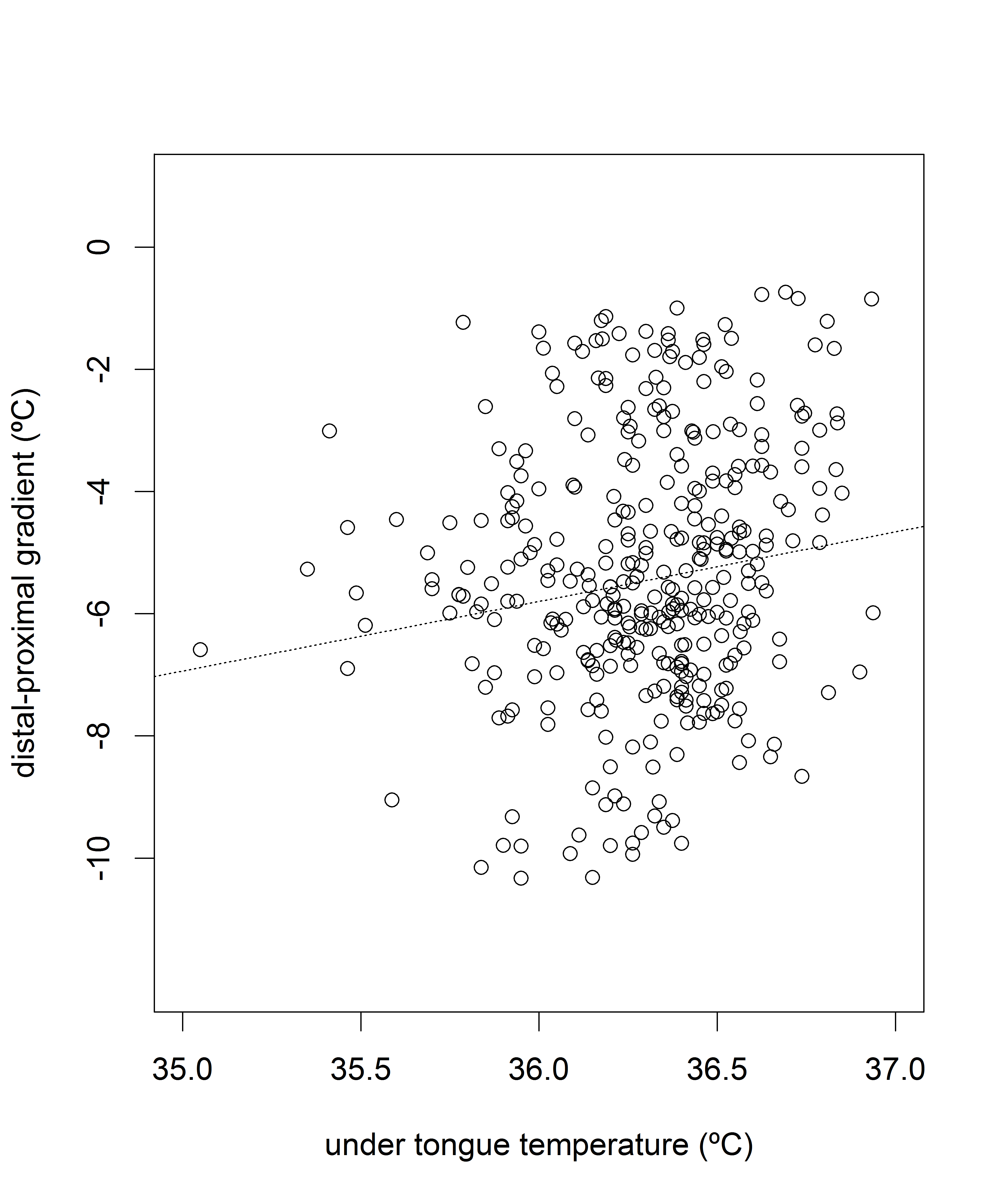
**

**Figure S4.** Relationship between temperature measured underneath the tongue and distal-proximal gradient. A significant positive correlation between temperature measured under the tongue and distal-proximal gradient could be detected (p=0.0001).

***T_tongue_ as proxy for CBT.*** Temperature variations were measured with skin temperature measurements on various proximal and distal locations as well as underneath the tongue. CBT is usually measured rectally, tympanic or esophageal, and is relatively costly and/or invasive. Variation in T_tongue_ resembled variation in T_collarbone_ and T_navel_, which are thought to be skin temperature proxies for CBT. Furthermore, correlations between T_tongue_ and distal proximal gradient exist, which reveal a similar pattern as found in literature between CBT and DPG (Cagnacci et al., 1997). Taken together, these are strong indications that T_tongue_ can be considered to resemble CBT. More importantly, T_tongue_ is likely to resemble brain temperature, which can be more relevant for cognitive performance.

**References.**

Cagnacci, A., Krauchi, K., Wirz-justice, A., & Volpe, A. (1997). Homeostatic versus Circadian Effects of Melatonin on Core Body Temperature in Humans. *J Biol Rhythm.*, *12*(6), 509–517.

Lucas, R. J., Peirson, S. N., Berson, D. M., Brown, T. M., Cooper, H. M., Czeisler, C. A., Figueiro, M. G., Gamlin, P. D., Lockley, S. W., O’Hagan, J. B., Price, L. L. A., Provencio, I., Skene, D. J., & Brainard, G. C. (2014). Measuring and using light in the melanopsin age. *Trends Neurosci.*
